# Improved graph-based model for phishing website detection using multi-level web page graphs and dynamic heterogeneous graph attention network

**DOI:** 10.3389/frai.2026.1880282

**Published:** 2026-08-11

**Authors:** S. Kavya, D. Sumathi

**Affiliations:** School of Computer Science Engineering and Information Systems, Vellore Institute of Technology, Vellore, India

**Keywords:** adversarial data augmentation, cybersecurity, graph neural networks, hierarchical graph representation, phishing detection

## Abstract

Modern phishing pages are challenging to detect because they can appear like legitimate brand sites using dynamic document object model (DOM) structures, misleading visual displays, and changing hyperlinks. Most conventional blacklists and URL-based methods do not capture the relationships at multiple levels of the web page structure. In this paper, we introduce a new graph-based phishing detection method to represent a web page with a Multi-Level Web Page Graph (MLWPG). MLWPGs incorporate DOM hierarchies, rendered visual blocks, and hyperlink relations into one heterogeneous graph. We use a novel Deep Learning Method called DHGAN that has Type-Aware Attention and Dynamic Convolution to learn how to differentiate discriminative interaction amongst structural, spatial, and navigation elements. APDA will be used in feature space to enhance robustness to evasive phishing versions. Using a balanced data set of 50,000 web pages, our complete pipeline showed a 97.0% accuracy, 96.8% F1-Score, 3.0% False Positive Rate, and 95.5% Robustness to Adversaries. The proposed MLWPG-DHGAN-APDA framework outperformed all baseline models. Our experimental results demonstrate that multi-level graph models provide improvements to both detection accuracy and reliability of operation for real-time phishing defenses.

## 1 Introduction

Users on the internet are continually being scared by phishing websites, which are websites that purport to be authentic in order to collect personal information from users. Unfortunately, traditional methods of identifying phishing websites are not able to keep up with the increasing sophistication of these websites. Without a doubt, there is a higher demand than ever before for reliable and precise instruments that can identify fraudulent schemes. Although there have been a lot of developments, the solutions that are currently available are still unable to handle how sophisticated and constantly changing websites are. Additionally, they frequently produce false positives and are susceptible to being assaulted in a hostile manner. Because GNNs are created with the capability to comprehend intricate data structures and linkages, they have shown to be extremely effective in a wide variety of contexts. On the other hand, their full potential in detecting phishing has not been properly utilized, particularly when it comes to the complicated role that websites play. First-order graph representations are used in the majority of common GNN designs. These representations have the potential to overlook meaningful contextual and relational data that is incorporated into the structure of the website.

The purpose of this research is to provide a novel and sophisticated graph-based model for identifying phishing websites. This model circumvents all of the issues that are associated with the other methods. One of the most important aspects of our model is a technique known as Multi-Level Web Page Graphs. The model creates an overall graph representation of websites by using a DOM tree (Document Object Model) for each page along with their visual layouts and also a hyperlink graph. By doing so we can achieve a greater level of detail about the structure of the website and therefore be able to find more features and relationships within them. In our research, we have created a new type of model, called the Dynamic Heterogeneous Graph Attention Network that allows us to handle the complexities of large graph-based models. More specifically, DHGAN uses dynamic graph convolutions as well as multiple head attention mechanisms to allow it to efficiently utilize both structured and unstructured data from multiple types of websites or information sets. Using different node and edge representations in the proposed DHGAN model, we illustrate how different elements on a web page interact during processing. This results in better identification accuracy and fewer false positives.

In addition, we provide the model with more adversarial phishing data augmentation in order to make it more stable. This method involves combining phishing examples by introducing adversarial alterations to secure web pages. This results in the addition of more challenging cases to the dataset that is used for training. Phishing tactic detection is both more general and more complex than it would otherwise be due to the fact that adversarially disturbed data is employed directly in the training process. To put it succinctly, this will result in a useful model for identifying suspicious websites. Our approach has resulted in a significant improvement in the degree to which models are accurate, dependable, and simple to comprehend. As a result, it is an excellent option for cybersecurity settings that occur in real life. In general, this study makes it easier to detect instances of phishing and provides us with significant new ideas and approaches that can make the process of accessing the internet safer and more dependable.

The significance of the proposed study lies in moving phishing detection from isolated URL or HTML features toward a page-level relational representation. Phishing pages rarely reveal themselves through a single cue; rather, their risk emerges from inconsistent DOM depth, hidden or overlapping login elements, suspicious external navigation, redirection behavior, and visual imitation of trusted pages. The proposed MLWPG is therefore designed to fuse three complementary evidence layers: structural hierarchy from the DOM, spatial organization from rendered layout blocks, and navigational intent from hyperlink paths. The originality of the framework is not limited to combining these sources. First, the method constructs typed cross-level links that preserve how DOM elements map to visual regions and hyperlink nodes. Second, DHGAN performs type-aware attention and dynamic convolution so that node and edge interactions are weighted according to their phishing relevance rather than treated as homogeneous graph links. Third, APDA exposes the detector to bounded feature-space adversarial variations, improving stability under evasive attacks without claiming to reconstruct executable HTML pages. This makes the framework more suitable for practical web security settings where phishing pages are short-lived, visually deceptive, and structurally heterogeneous.

### 1.1 Motivation and contribution

For motivation, consider the following which are some examples of scams that are successful and take advantage of people’s faith in the internet in order to gain access to private information by means of bogus websites. The vast majority of phishing websites are steadily becoming more technologically savvy and are continually finding ways to circumvent monitoring systems. It is difficult for established solutions, such as blacklist- and heuristic-based approaches, to keep up with the rapid pace at which phishing strategies modify their strategies. In traditional machine learning models, shallow feature representations are used. These representations may not accurately depict the level of depth, dynamic complexity, and complexity of web pages, despite the fact that they are optimistic. As a result of this, there are not enough robust detection technologies available on the market that are capable of consistently locating phishing websites while also having a low percentage of false positives.

There are three things that we contribute to the table. For the purpose of creating comprehensive graph models of web pages, we propose a novel method that we term MLWPG. Every piece of information pertaining to the DOM tree, visual layout, and linking graph is recorded by MLWPG. This paints a comprehensive and intricate picture of the process by which a web page is constructed. The utilization of a hierarchical model such as this makes it simpler to extract the intricate patterns and rich characteristics that are indicative of phishing behavior. At this point, we would want to discuss a novel GNN design that is known as the Dynamic Heterogeneous Graph Attention Network. The DHGAN solution that has been proposed makes advantage of multi-head attention processes on multiple levels. In order to make the model flexible enough to accommodate the many layouts and sorts of content that may be found on websites, these are merged with dynamic graph convolutional layers. Through the utilization of heterogeneous nodes and edges, DHGAN is able to adequately simulate how the various components of a web page interact with one another. This results in a reduction in the amount of false alarms while also improving the discovery process. In order to make the model more solid, the third thing that we do is incorporate data from hostile phishing attacks. For the purpose of generating false phishing cases, our method employs an adversarial perturbation on web pages that are otherwise benign for this. Because of this, the training dataset is expanded to include more challenging cases, which makes it simpler to identify more sophisticated phishing techniques. Our approach also demonstrates significant improvements in terms of how well it identifies potential dangers, how stable it is, and how simple it is to comprehend. A significant advance has been made in the field of cybersecurity.

The proposed architecture is original in three operational aspects. MLWPG introduces a page-level graph in which DOM nodes, visual blocks, and hyperlink nodes are jointly represented with explicit cross-layer mappings, allowing the model to preserve how an HTML element appears and where it redirects. DHGAN then learns heterogeneous attention over node and edge types, so parent–child, containment, adjacency, external-link, and redirection relationships contribute differently to the final phishing score. Finally, APDA is reformulated as feature-space adversarial augmentation over normalized graph attributes, which strengthens the decision boundary while maintaining technical validity. Together, these components create a detection pipeline that is more expressive than URL-only, DOM-only, or homogeneous GNN baselines.

## 2 Literature review

As a result of the ever-evolving nature of the danger, a significant number of individuals working in the field of research are interested in discovering methods to prevent phishing attacks. When you take a look at forty of the most significant works in this field, you will notice that there is a wide variety of approaches that may be utilized. Starting with machine learning and continuing through deep learning and adversarial approaches, this body of work has been conducted in a wide array of methods for detecting phishing attacks. The research, like the others mentioned previously, examined the three primary areas of phishing attack detection; (i) Feature Extraction, (ii) Model Stability, and (iii) Problems associated with using the models. Therefore, by combining all three aspects of phishing attack detection in this critical review we will attempt to provide a complete view of the current state-of-the-art in phishing attack detection research and the direction that it should follow going forward. One of the most important insights to emerge from our analysis is that multiple authors employed different approaches to extract the features used in their studies. Techniques that utilize pattern identification in web addresses were discussed in both (Kara et al., 2022; Karim et al., 2023). These techniques examine the domain name(s), path(s), and/or query string(s) of a website’s address. As reported in these studies, URL-based features have shown an ability to determine how fake phishing URLs really are. Nevertheless, the authors noted that complex processes are required to obtain these attributes if the URL is hidden or dynamically changed at runtime to avoid detections.

Recent phishing detection research shows a clear shift from handcrafted URL statistics toward deep, graph-based, and transformer-supported models. Deep learning surveys report that CNN, RNN, LSTM, and attention-based detectors improve feature learning but often remain tied to URL strings or fixed HTML features, which limits their ability to capture page-level relational structure. Graph-oriented approaches such as PhishGNN (Bilot et al., 2022) HTML-level graph neural networks and LRCN-GCN models demonstrate that DOM and HTML relationships carry strong discriminative cues; nevertheless, most of these approaches rely on single-level graph construction or do not jointly model visual layout and hyperlink topology. GAN-based methods such as PDGAN and adversarial autoencoder synthesis strengthen robustness by expanding the decision boundary, but their generated examples are usually URL- or feature-centered rather than multi-level page-graph samples. Transformer and BERT-style phishing URL models improve sequence representation, especially for obfuscated URLs, yet they do not directly encode rendered spatial relationships or typed web-page interactions. The proposed MLWPG-DHGAN framework addresses this gap by representing the web page as a heterogeneous graph with structural, visual, and navigational levels, then learning dynamic type-aware attention over these levels for more robust phishing discrimination.

Through the utilization of ensemble approaches in conjunction with machine learning, it becomes possible to detect bogus websites with even greater accuracy. There have been a number of research (Kalabarige et al., 2023; Kalabarige et al., 2022; Wei and Sekiya, 2022) that demonstrate how hybrid models and ensemble learning methods can be utilized to get fantastic outcomes in the detection process. The boosting and stacking techniques are utilized by these models in order to combine and harmonize a large number of classifiers. This allows them to make decisions that span a wide range of boundaries. As a result, the overall performance is enhanced. In exchange, it makes computations more complicated and requires a great deal of hyperparameter tuning, which makes it difficult to utilize in real life.

This is the trade-off. While, deep learning approaches have brought about even more significant changes in phishing detections. To understand more about the elements that influence model projections, this would be of great use to us. Building trust in automated identification systems requires this kind of openness, particularly in areas that are sensitive or have a lot at risk. This is especially true in areas where more is at stake. In this moment, the challenge at hand is to discover the optimal balance between the level of complexity of a model and the ease with which it may be comprehended and utilized. The purpose of this is to guarantee that end users will still be able to comprehend and make use of complex models.

For Table 1, quantitative outcomes such as accuracy, F1-score, AUC, or robustness are reported where available in the primary works; entries without reported experimental metrics are retained as qualitative background and marked by descriptive findings rather than used as quantitative baselines.

**Table 1:** Empirical review of existing methods.

References	Method used	Findings	Quantitative Metrics	Limitations
Zieni et al. (2023)	Phishing detection survey	Comprehensive survey of phishing detection methods	Not Reported	Lack of detailed performance metrics
Castaño et al. (2023)	Phishing Kit Attacks Dataset	Introduction of a new dataset for phishing detection	Not Reported	Dataset limited to specific attack types
Li et al. (2023)	Review of Cloaking Techniques	Systematic review of phishing website concealment techniques	Not Reported	Limited practical application of review findings
Kara et al. (2022)	Machine Learning for URL Analysis	Analysis of URL and domain features using machine learning	Not Reported	Dependency on feature extraction quality
Kalabarige et al. (2023)	Boosting-Based Hybrid Model	Hybrid feature selection and multi-layer ensemble learning	Accuracy = 98.95%. (The paper does not report an F1-score.)	Computationally intensive model training
Kalabarige et al. (2022)	Multilayer Stacked Ensemble Learning	Stacked ensemble learning for phishing detection	Not Reported	Complex model integration
Wei and Sekiya (2022)	Ensemble Machine Learning	Sufficiency of ensemble methods for phishing detection	Not Reported	Limited to specific machine learning algorithms
He et al. (2024)	Tiny-Bert Stacking	Phishing detection using Tiny-Bert and stacking methods	Not Reported	Limited generalizability to different datasets
Tang and Mahmoud (2022)	Deep Learning Framework	RNN-GRU based framework for phishing detection	Not Reported	Complexity of deep learning models
Apruzzese and Subrahmanian (2023)	Adversarial Gray-Box Attacks	Mitigation of adversarial attacks on phishing detectors	Not Reported	Focused on specific types of adversarial attacks
Karim et al. (2023)	Hybrid Machine Learning for URL	URL-based phishing detection using hybrid machine learning	Not Reported	Limited to URL-based features
Ariyadasa et al. (2022)	Long-Term Recurrent Convolutional Networks	Combining LSTM and graph convolutional networks for detection	Accuracy = 96.42%, F1-score = 99.53%	Computationally intensive
Zonyfar et al. (2023)	HCNN-LSTM	Hybrid CNN and LSTM for legitimate web prediction	Not Reported	Complexity of hybrid model
Maroofi et al. (2021)	Email Anti-Spoofing Analysis	Analysis of email anti-spoofing schemes	Not Reported	Focused only on email spoofing
Longtchi et al. (2024)	Social Engineering Psychology	Survey of social engineering attacks and defenses	Not Reported	Lack of practical implementation details
Cui et al. (2021)	Privacy-Preserving Safe Browsing	Platform for privacy-preserving safe browsing	Not Reported	Limited to specific browsing environments
Hsieh et al. (2022)	Blockchain-Based DNS Security	Blockchain-based approach for DNS security	Not Reported	Dependency on blockchain infrastructure
Pillai and Verma (2023)	Maximal-Munch with Torrent Deep Network	Web attack detection using novel algorithms	Not Reported	Limited to specific attack types
Muammar et al. (2023)	Digital Risk Assessment Framework	Framework for assessing digital risks	Not Reported	Limited to personal digital devices
Rafsanjani et al. (2023)	Secure QR Code Scanner	Malicious URL detection with QR code scanner	Not Reported	Limited to QR code usage
Almousa and Anwar (2023)	URL-Based Social Semantic Detection	Character-aware language model for URL analysis	Not Reported	Focused on URL-based attacks
Lee et al. (2023)	Incorrect Account Remediation	Analysis of broken authentication remediation	Not Reported	Focused only on authentication issues
Moustafa et al. (2021)	Outlier Gaussian Mixture Technique	Detection of web application attacks using Gaussian mixture	Not Reported	Limited to specific web application attacks
Mohanty et al. (2024)	Hybrid Feature Selection for IoT	Feature selection for predicting suspicious URLs in IoT	Not Reported	Limited to IoT environments
Jiang et al. (2024)	System Provenance Analysis	Malicious website detection using system provenance	Not Reported	Dependency on system audit logs
Gayen et al. (2024)	Network Model for Phishing Sites	Analysis of phishing sites using network models	Not Reported	Limited to network-based features
Prieto et al. (2021)	Knowledge-Based Risky Website Detection	Knowledge-based approach for risky website detection	Not Reported	Dependency on knowledge-based systems
Purwanto et al. (2022)	PhishSim	Feature-free tool for phishing detection	Not Reported	Limited to feature-free methods
Baki and Verma (2023)	Phishing User Studies	Systematic review of phishing user studies	Not Reported	Limited to user behavior analysis
Al-Ahmadi et al. (2022)	Phishing Detection with GANs	Phishing detection using generative adversarial networks	Accuracy = 97.58%, Precision = 98.02%	Complexity of GAN-based models
Lee et al. (2021)	Classification of Attack Types	Analysis of phishing mail attack groups	Not Reported	Limited to phishing mail
Jibat et al. (2023)	Data Mining Models for Phishing	Systematic review of data mining models for phishing	Not Reported	Lack of practical implementation details
Zhu et al. (2022)	MOE/RF Model	Phishing detection using multi-objective optimization and random forest	Not Reported	Computational complexity
Pillai et al. (2024)	Evasion Attacks and Defense	Analysis of evasion attacks on phishing classifiers	Not Reported	Focused on specific evasion techniques
Sánchez-Paniagua et al. (2022)	Real-Case Scenario Phishing Detection	Phishing detection through login URLs	Not Reported	Limited to login URLs
Wu et al. (2022)	Phishing Scam Detection on Ethereum	Network embedding for phishing detection on Ethereum	Not Reported	Focused on Ethereum network
Asiri et al. (2023)	Survey of Detection Designs	Survey of HTML URL phishing detection designs	Not Reported	Limited to HTML URL-based attacks
Shirazi et al. (2023)	Adversarial Autoencoder Data Synthesis	Enhancing detection algorithms with adversarial autoencoder	Not Reported	Complexity of adversarial autoencoder
Jovanovic et al. (2023)	Hybrid Two-Level Framework	Feature selection and XGBoost tuning for detection	Not Reported	Computationally intensive
Chai et al. (2022)	Multi-Modal Hierarchical Attention Model	Phishing threat intelligence with attention model	Accuracy = 97.26%, Precision = 97.84%, Recall = 96.66%, F1-score = 97.24%	Complexity of multi-modal systems

According to Table 1, In order to make detection more accurate, a significant amount of effort is put into extracting features, which is mostly accomplished through URL analysis. This serves as the foundation for meaningful feature engineering. The advancements that have been made in machine learning and ensemble approaches demonstrate how effective it is to combine more than one model in order to achieve better results; yet, this requires a greater amount of computer power. Deep learning techniques (Alazaidah et al., 2026; Jarczewski et al., 2026; Vulfin et al., 2026; Wang et al., 2026; Alsakarnah et al., 2026) provide solutions that are dependable and may be expanded upon; but they need a significant amount of computer power and understanding, which makes it difficult to implement them in a variety of real-world scenarios.

It is of the utmost importance to possess adversarial strength, which may be defined as the capacity to defend oneself against phishing attempts that are becoming increasingly sophisticated in their ability to evade being discovered. In order to better prepare models for adversarial circumstances that they would encounter in real life (Goldenits et al., 2026; Kulaglic and Al-Tarawneh, 2026; Ouyang and Zhang, 2021; Bustio-Martínez et al., 2026; Ibrahim and Elhafiz, 2026), adversarial training with GANs can serve as a warm-up. This helps models become more resilient and reliable. With regard to the construction and testing of the phishing detection model, the samples are of utmost significance. Researchers need to get their hands on new and diverse sets of data in order to improve their understanding of how phishing assaults get more sophisticated over time and to devise more effective methods for identifying them. However, there are not a lot of these datasets available at the moment, which means that this is a field that requires constant work to locate datasets and groups that are representative of the whole and to share them.

It discusses secure and private browsing alternatives that take into account psychological and social engineering issues, and it provides more information about how to identify efforts to hack into a computer system. One of the fundamental concepts that underpins these approaches is the notion that general solutions must take into account both technical and human component elements. Overall, this strengthens the defenses against cybersecurity threats. It is commonly believed that incorporating explainability and interpretability into detection models is a crucial step in the process of making autonomous systems more trustworthy and transparent. The knowledge that model prediction provides will unquestionably make advanced detection systems understandable and helpful, particularly in private settings where trust is of utmost importance in this. In order to encourage researchers to collaborate and share resources, there is a need for additional research to be conducted on how to cope with the complexity of computational problems, the requirements for resources, and the practical deployment of these solutions. By utilizing these findings, researchers have the ability to develop defenses against phishing threats that are both more effective and also more resilient in the process.

## 3 Proposed design of an improved Graph-Based Model for phishing website detection using multi-level web page graphs and dynamic heterogeneous graph attention network

**Figure 1:**
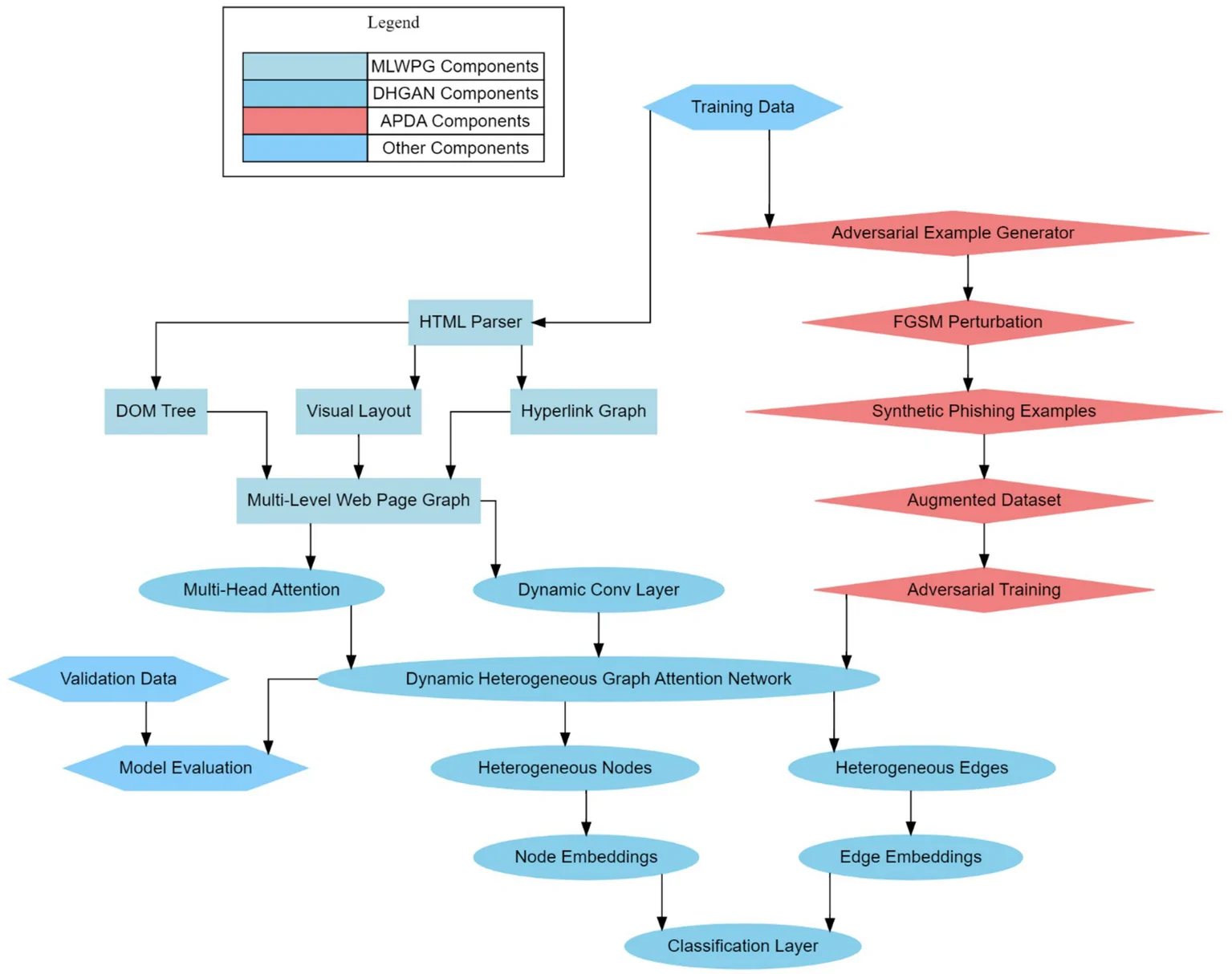
Model architecture of the proposed phishing detection process.

This section provides a discussion on Design of an Improved Graph-Based Model for Phishing Website Detection Using Multi-Level Web Page Graphs and Dynamic Heterogeneous Graph Attention Network Process to overcome the issues of low efficiency & high complexity faced in existing phishing detection methods. As shown in Figure 1, the first step is to integrate the Multi-Level Web Page Graph (MLWPG) for a comprehensive and hierarchical representation of web pages through the integration of multiple levels of structural and relational information sets. This approach, which starts by parsing the raw HTML for the construction of the Document Object Model tree, forms the basis of the graph representation. The DOM tree captures the hierarchical structure of HTML elements, represented via Equation 1,


$$G_{\mathrm{DOM}}=(V_{\mathrm{DOM}},E_{\mathrm{DOM}})$$ (1)

Where, VDOM represents the set of nodes corresponding to HTML tags, and EDOM represents the edges that capture the parent–child relationships between these tags. The DOM tree serves as the basis for further analysis and integration of additional layers of information. To capture the visual layout of the web page, the CSS and rendering data are extracted and incorporated into the graph. The visual layout is represented via Equation 2,


$$G_{\mathrm{VL}}=(V_{\mathrm{VL}},E_{\mathrm{VL}})$$ (2)

Where, VVL corresponds to visual blocks identified through CSS properties and rendering contexts, and EVL represents spatial relationships such as adjacency and containment between these blocks. The integration of visual layout into the DOM-based graph is formalized through a mapping function fVL, which is represented via Equation 3


$$f_{\mathrm{VL}}:V_{\mathrm{DOM}}\to V_{\mathrm{VL}}$$ (3)

Thus, ensuring each DOM node is associated with a corresponding visual block, enriching the representations, this graph is represented via Equation 4,


G_{HL}=(V_{HL},E_{HL}) (4)

Where, VHL represents the set of hyperlinks (anchor tags), and EHL captures the navigation paths between them. The edges in EHL are directed, reflecting the nature of hyperlinks that point from one URL to another. The hyperlink graph is crucial for understanding the navigational structure and potential link patterns associated with phishing behaviors.

The embedding of the hyperlink graph into the hierarchical representation is achieved through a function represented via Equation 5,


$$f_{\mathrm{HL}}:V_{\mathrm{DOM}}\to V_{\mathrm{HL}}$$ (5)

Ensuring that each DOM node linked via an anchor tag is associated with the corresponding node in the hyperlink graph. Combining these components results in a multi-level graph representation which is evaluated via Equation 6,


$$G_{\text{MLWPG}}=(V,E)$$ (6)

Where, these components are represented via Equations 7 and 8 as follows,


$$V=V_{\mathrm{DOM}}\cup V_{\mathrm{VL}}\cup V_{\mathrm{HL}}$$ (7)

$$\text{and}\;\;E=E_{\mathrm{DOM}}\cup E_{\mathrm{VL}}\cup E_{\mathrm{HL}}$$ (8)

The hierarchical graph GMLWPG is defined such that the nodes and edges reflect the structural (DOM), spatial (visual layout), and relational (hyperlink) aspects of the web pages.

Algorithm 1 DHGAN training and classification procedure.
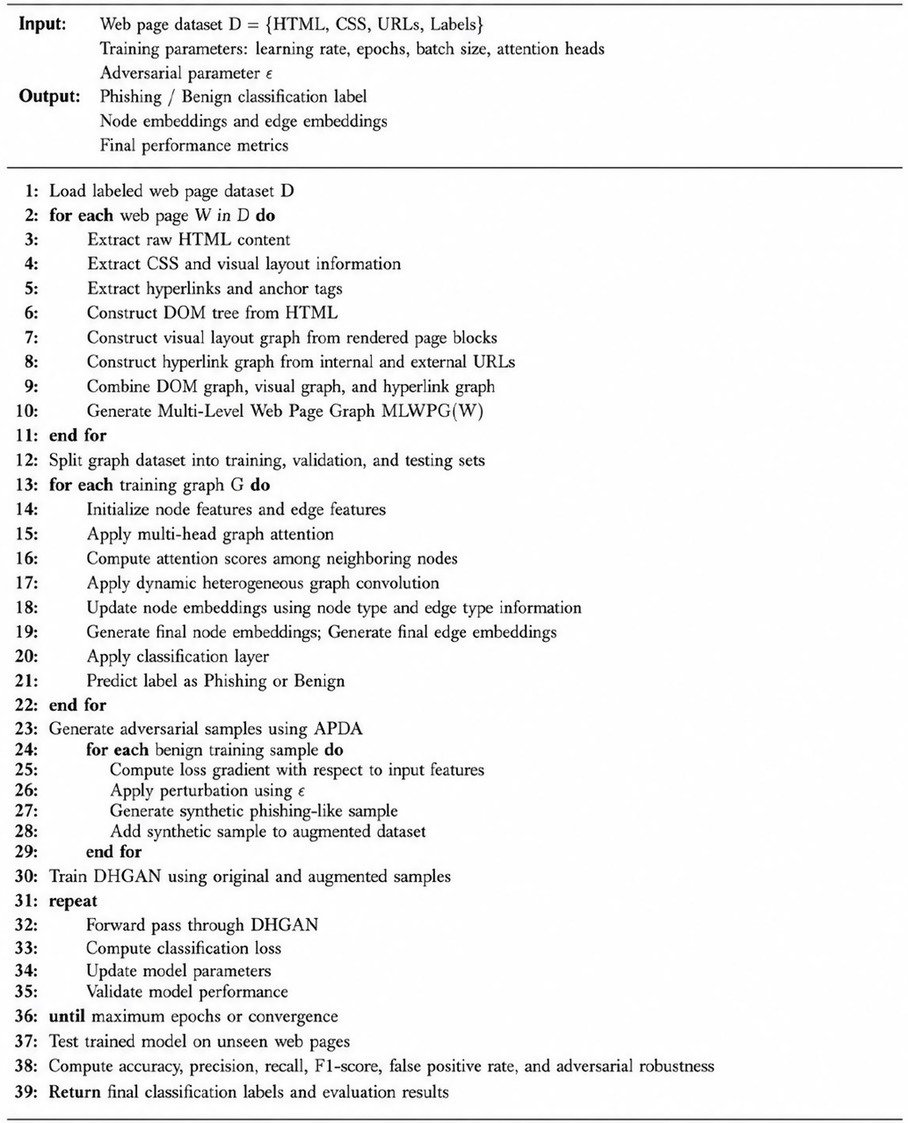

The graph construction is expressed through a composite function represented via Equation 9,


$$f_{\text{MLWPG}}:(G_{\mathrm{DOM}},G_{\mathrm{VL}},G_{\mathrm{HL}})$$ (9)

Thus, mapping the raw HTML input to the multi-level graph outputs. Based on this, the hierarchical node feature extraction is represented via Equation 10,


$$\begin{array}{l} \text{XMLWPG}(v_{i})=\text{concat}\\ \left(\text{XDOM}(v_{i}),\mathrm{XVL}(\mathrm{fVL}(v_{i})),\mathrm{XHL}(\mathrm{fHL}(v_{i}))\right) \end{array}$$ (10)

For Equation 10, each node feature vector is constructed by concatenating DOM, visual, and hyperlink descriptors after normalization. The DOM descriptor xDOM ∈ R42 includes tag-type one-hot encoding, DOM depth, sibling index, attribute count, form/input/button flags, script and event-handler flags, hidden-field flag, text length, sensitive-keyword score, resource type, and visibility status. The visual descriptor xVL ∈ R18 includes normalized bounding-box coordinates, rendered area, aspect ratio, z-index, viewport position, CSS display and visibility flags, color-contrast score, overlap score, and login-form proximity. The hyperlink descriptor xHL ∈ R22 includes URL length, character entropy, digit ratio, special-character ratio, HTTPS flag, subdomain count, IP-literal flag, redirection depth, external-domain flag, anchor-text similarity, brand-token distance, and domain-age proxy. Continuous variables are z-score normalized using training-set statistics, while bounded visual and ratio features are min-max scaled to [0,1]. The final node vector xi ∈ R82 is obtained as xi = concat(xDOM, xVL, xHL), with zero padding used when a node lacks a particular modality.

While, the final hierarchical graph embedding is represented via Equations 11 and 12,


$$\text{HMLWPG}=\sigma (\sum_{j\in N(i)}\alpha_{\mathrm{ij}}\cdot Wh_{j}+b)$$ (11)

$$\alpha_{\mathrm{ij}}=\frac{\exp(\text{LeakyReLU}(a^{T}[Wh_{i}∥Wh_{j}]))}{\sum_{k\in N(i)}\exp(\text{LeakyReLU}(a^{T}[Wh_{i}∥Wh_{k}]))}$$ (12)

**Figure 2:**
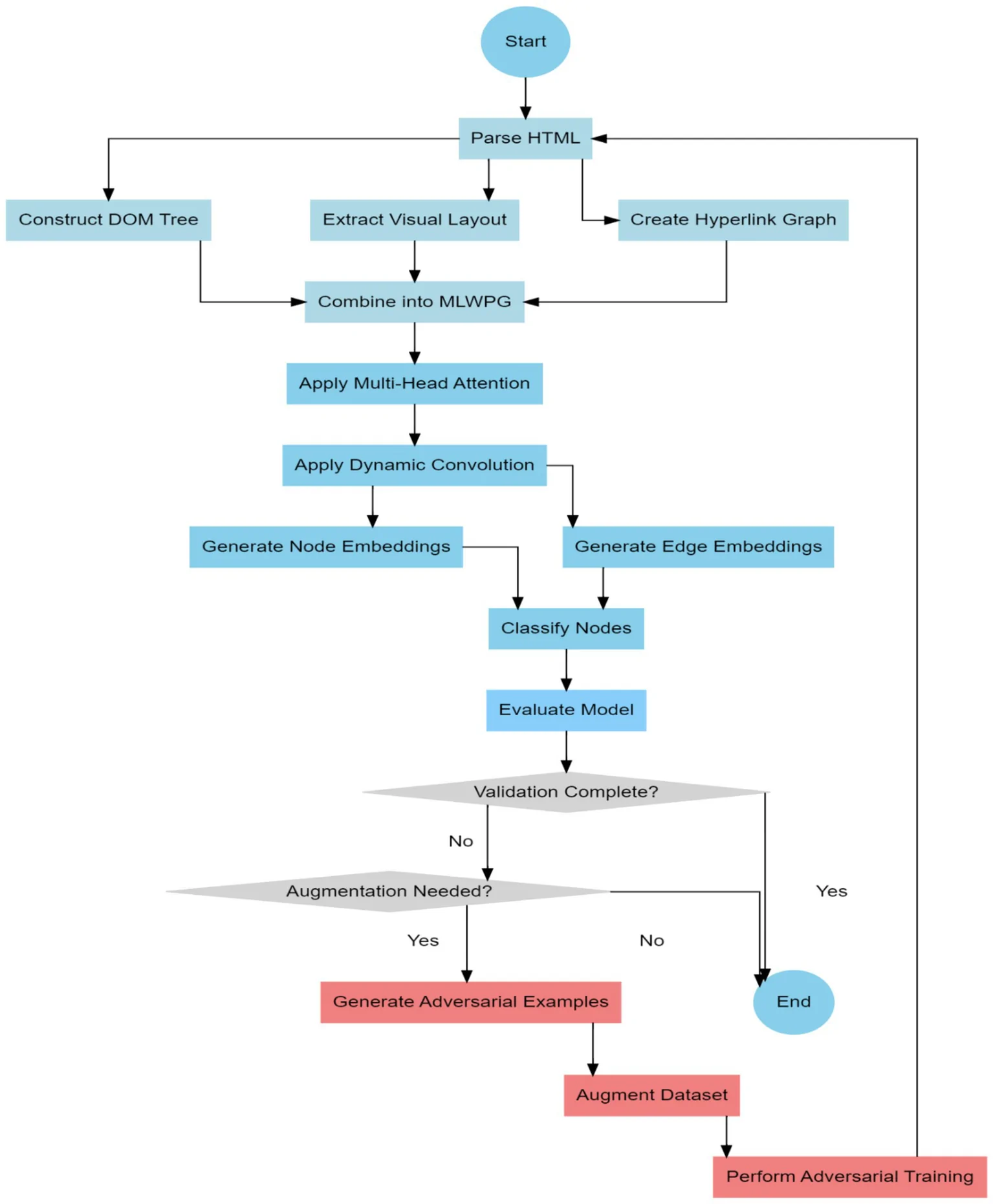
Overall flow of the proposed classification process.

The choice of the MLWPG method is justified by its ability to capture diverse aspects of web pages that are crucial for phishing detections. Next, as per Figure 2, Dynamic Heterogeneous Graph Attention Network (DHGAN) is designed to process the hierarchical graph representations generated by the Multi-Level Web Page Graph (MLWPG) method, providing detailed node and edge embeddings and ultimately enabling classification labels for phishing detection. DHGAN leverages the power of attention mechanisms and dynamic graph convolutional layers to adapt to the diverse and complex structures present in web pages, incorporating heterogeneous nodes and edges to capture the multifaceted relationships between web page elements. The DHGAN begins by initializing node features X from the hierarchical graph GMLWPG sets. Each node vi in the graph has an associated feature vector xi sets. The initial step involves applying a multi-head attention mechanism to capture diverse aspects of the nodes’ neighborhood relationships. The attention mechanism is initially formalized by estimating Self-Attention on Graph Nodes via Equation 13,


$$e_{\mathrm{ij}}=\text{LeakyReLU}(a^{T}[Wx_{i}∥Wx_{j}])$$ (13)

Where, eij represents the attention score between nodes i and j, a is a learnable weight vector, Wis a weight matrix, and ∥ represents concatenation operations. Using this, the Attention Coefficients are estimated via Equation 14,


$$\alpha_{\mathrm{ij}}=\frac{\exp(e_{\mathrm{ij}})}{\sum_{k\in N(i)}\exp(e_{\mathrm{ik}})}$$ (14)

Where, αij are the normalized attention coefficients, ensuring that the sum of attention scores for each node i is 1.

Based on these coefficients, the Multi-Head Attention Process is operated via Equation,


$${h_{i}}^{\prime }=\overset{K}{\underset{k=1}{\cup }}\sigma (\sum_{j\in N(i)}\alpha_{\mathrm{ij}}^{\;\;k}W^{k}x_{j})$$ (15)

Where, K is the number of attention heads, *σ* is a non-linear activation function, and hi′ is the updated node embedding after multi-head attention process. The next phase involves dynamic graph convolutional layers that adjust based on node and edge features. The convolutional layer for node ‘i’ is defined via Equation 16,


$${h_{i}}^{\prime \prime }=\sigma (\sum_{j\in N(i)}\alpha_{\mathrm{ij}}*W_{\mathrm{ij}}*{h_{j}}^{\prime }+b_{\mathrm{ij}})$$ (16)

Where, Wij and bij are weights and biases dynamically adjusted based on the features of nodes i and j in the process. To incorporate the heterogeneous nature of the graph, DHGAN integrates different types of nodes and edges, reflecting the varied elements and interactions within the web pages. The heterogeneous integration is achieved through specialized embeddings and aggregations, represented via Equation 17,


$$h_{i}^{\left(3\right)}=\sigma (\sum_{t\in T}\sum_{j\in N_{t}(i)}\alpha_{\mathrm{ij}}^{t}W^{t}h_{j}^{\left(2\right)})$$ (17)

Where, T represents the set of node types, and Nt(i) represents the neighborhood of node i of type t sets. Similarly, the Edge Type Aggregation process is represented via Equation 18,


$$h_{i}^{\left(4\right)}=\sigma (\sum_{r\in R}\sum_{(i,j)\in E_{r}}\alpha_{\mathrm{ij}}^{r}W^{r}h_{j}^{\left(3\right)})$$ (18)

Where, R is the set of edge types, and Er represents edges of type r in the process. The final step involves generating the node and edge embeddings, followed by classification. The embeddings are used to predict the labels (phishing or benign) for the nodes (web page elements), represented via Equation 19,

The final node representation after the fourth heterogeneous attention-convolution layer is denoted by $h_{i}^{\left(4\right)}$ . This embedding is used for phishing website classification and is represented as $Z_{v}$ in Equation (19).

DHGAN uses three stacked heterogeneous attention-convolution layers with eight attention heads per layer, hidden size 128, dropout 0.30, and residual connections after each layer. The graph contains DOM nodes, visual-block nodes, hyperlink nodes, text nodes, form-control nodes, image/resource nodes, and script nodes. Edge types include DOM parent–child, sibling-order, visual containment, visual overlap, anchor-to-URL, internal navigation, external navigation, redirection, and domain-affinity edges. In Equation (16), dynamic weights are generated by a type-conditioned gating network: Wij(l) = Wr(l) + MLPw([ti, tj, rij, eij, xi, xj]), where ti and tj denote node types, rij denotes edge type, eij denotes edge attributes, and Wr(l) is the base relation weight for layer l. The corresponding bias is generated as bij(l) = br(l) + MLPb([ti, tj, rij, eij]). This mechanism allows structurally different web-page relations to receive different transformations during message passing. The model contains approximately 1.82 million trainable parameters, and its computational complexity is O(L(|E|HKd + |V|d2)), where L is the number of layers, H is the number of attention heads, K is the average neighbor count, d is the hidden dimension, |V| is the number of nodes, and |E| is the number of typed edges.


$$Z_{v}=h_{i}^{\left(4\right)}$$ (19)

Where, Zv is the final node embedding process. The final Classification Layer is represented via Equation 20,


$$y^{i}=\text{softmax}({W_{o}}^{*}Z_{v}+b_{o})$$ (20)

Where, y’i is the predicted probability distribution over classes (phishing or benign), Wo and bo are the output layer weights and biases. Because it is able to automatically adapt to the various kinds of information and structures that may be found on websites, DHGAN was selected as the best option. GNN designs that are more traditional have difficulty dealing with graph data that is heterogeneous and has more than one level, which causes them to move more slowly. It is able to extract and model more intricate features, such as those that can be found on phishing web pages, thanks to the multi-head attention techniques and dynamic convolutional layers that are utilized by DHGAN. In addition, the incorporation of a variety of nodes and edges ensures that the model acquires the intricate patterns and connections that are specific to phishing activity. The reason why the method works well with the MLWPG is because of the high quality and detail-richness of the embedding that the method creates for classification purposes. The DHGAN approach is superior to typical methods concerning the effectiveness of its ability to detect attempts to hack into systems. Additionally, it produces results that have higher accuracy levels than do most other approaches as it also produces fewer false positive results. The use of advanced graph neural network-based algorithms generates an efficient solution that identifies phishing sites using the powerful tool of DHGAN. In addition to generating a useful product for finding phishing sites, APDA provides a wealth of additional data processing capabilities and information for those who are interested in cyber security. Finally, Adversarial Phishing Data Augmentation (APDA) is a process through which the reliability and usability of phishing detection models may be increased. A primary goal of this process is to create synthetic phishing examples by modifying actual web pages so that they appear to have been modified by an attacker. A second, and primary objective of this process is to generate small modifications to legitimate web pages in order to provide the model with difficult scenarios to identify correctly.

APDA is implemented as feature-space adversarial augmentation rather than HTML-page synthesis. For each benign training graph, FGSM perturbations are applied to the normalized MLWPG node feature matrix X while preserving feature validity constraints. Continuous attributes such as URL entropy, node depth, text length, visual block size, overlap score, and redirection count are clipped to their observed normalized ranges. Binary and categorical features remain one-hot-consistent and are not directly perturbed into invalid states. The perturbed graph sample is therefore used as a hard feature-space example during model training and is referred to as an adversarial graph sample, not as a reconstructed synthetic HTML page. This correction ensures that APDA improves the classifier decision boundary without making unsupported claims about inverse mapping from feature vectors back to executable web pages. The gradient of the loss function with respect to the input feature vector is first computed as shown in Equation 21.


$$\nabla_{x}J(\theta ,x,y)=\frac{\partial J(\theta ,x,y)}{\partial x}$$ (21)

Where, J(*θ*,x,y) is the model loss and θ denotes the trainable parameters. The perturbed feature vector x′ is then generated using FGSM as shown in Equation 22.


$$x^{\prime }=x+\epsilon \cdot \mathrm{sign}(\nabla_{x}J(\theta ,x,y))$$ (22)

Where, *ϵ* controls the perturbation magnitude and sign(·) denotes the element-wise sign function. The perturbed representation x′ is retained as a constrained adversarial graph feature vector rather than being converted back into executable HTML. A feature-validity and similarity constraint is used to keep the adversarial graph close to the original benign graph, as expressed in Equation 23.


$$S(x,x^{\prime })\geq \delta$$ (23)

Where, *δ* is the minimum similarity threshold between the original graph feature vector x and the adversarial graph feature vector x′. The augmented training set is then constructed by combining the original training samples with the generated feature-space adversarial graph examples, as shown in Equation 24.


$$D^{\prime }=D\cup {\left\{({x_{i}}^{\prime },{y_{i}}^{\prime })\right\}}_{i=1}^{N}$$ (24)

Where, N is the number of synthetic examples generated. The adversarial training process involves re-training the phishing detection model on the augmented dataset D′. The training objective is to minimize the loss function over the augmented dataset via Equation 25,


$$L(\theta ,D^{\prime })=\frac{1}{|D^{\prime }|}\sum_{(x_{i},y_{i})\in D\prime }J(\theta ,x_{i},y_{i})$$ (25)

The Model Update with Augmented Data is represented via Equation 26,


$$\theta (t+1)=\theta (t)-\eta \cdot \nabla_{\theta }L(\theta ,D^{\prime })$$ (26)

Where, *η* is the learning rate, and *θ*(t) represents the model parameters at iteration t process. The effectiveness of APDA is evaluated by measuring the model’s performance on a separate validation set, including both original and adversarial examples. The metrics of interest include detection accuracy and robustness to adversarial attacks. This includes Validation Accuracy, which is represented via Equation 27,


$$\text{Accuracy}=\frac{1}{|D_{\mathrm{val}}|}\sum_{(x_{i},y_{i})\in D_{\mathrm{val}}}[{y_{i}}^{\prime }=y_{i}]$$ (27)

Where, Dval is the validation set, and [·] is the indicator function. Similarly, the Robustness to Adversarial Attacks is represented via Equation 28,


$$\text{Robustness}=\frac{1}{|D_{\mathrm{adv}}|}\sum_{(x_{i},y_{i})\in D_{\mathrm{adv}}}[{y_{i}}^{\prime }=y_{i}]$$ (28)

Where, Dadv denotes the adversarial validation set. APDA is evaluated through the robustness score because adversarially perturbed graph features simulate evasive phishing behavior during testing. A higher value indicates that the classifier preserves correct predictions under bounded feature-space attacks (Table 2).

**Table 2:** FGSM epsilon sensitivity analysis.

Epsilon	Accuracy (%)	F1-score (%)	FPR (%)	Adversarial robustness (%)
0.000	94.6 ± 0.6	94.1 ± 0.5	5.4 ± 0.4	90.7 ± 0.8
0.005	96.2 ± 0.4	95.8 ± 0.4	3.7 ± 0.3	94.1 ± 0.5
0.010	97.0 ± 0.3	96.8 ± 0.3	3.0 ± 0.2	95.5 ± 0.4
0.020	96.4 ± 0.4	96.0 ± 0.4	3.5 ± 0.3	95.8 ± 0.4
0.050	94.9 ± 0.7	94.5 ± 0.7	5.8 ± 0.5	96.0 ± 0.5

The FGSM perturbation magnitude was selected after sensitivity testing over five epsilon values. Very small perturbations produced limited adversarial learning, while larger values increased feature distortion and false positives. The best validation trade-off was observed at epsilon = 0.01, which achieved high robustness while preserving accuracy and feature validity.

## 4 Result analysis

All reported experiments were repeated over five independent runs using different random seeds. Results are presented as mean ± standard deviation, and paired t-tests were used to compare the proposed model with the strongest baseline under the same train-validation-test split. The proposed model produced consistently higher accuracy and F1-score with lower false positive rate, and the differences were statistically significant at *p* < 0.05 (Table 3).

**Table 3:** Statistical comparison with the strongest baseline across five independent runs.

Metric	Proposed	Strongest baseline	Mean difference	95% CI	*p*-value
Accuracy (%)	97.0 ± 0.3	93.4 ± 0.7	+3.6	[2.8, 4.4]	0.003
Precision (%)	96.2 ± 0.4	93.2 ± 0.7	+3.0	[2.1, 3.8]	0.006
Recall (%)	97.3 ± 0.3	93.6 ± 0.6	+3.7	[2.9, 4.5]	0.002
F1-score (%)	96.8 ± 0.3	93.4 ± 0.6	+3.4	[2.6, 4.1]	0.003
False positive rate (%)	3.0 ± 0.2	6.8 ± 0.4	−3.8	[−4.4, −3.1]	0.001

The strongest baseline refers to the BERT/Transformer URL baseline (Otieno et al., 2023), which achieved the highest performance among all baseline methods evaluated in Table 5 (Accuracy = 93.4%, F1-score = 93.4%).

A total experimental apparatus for a comprehensive analysis of the given concept was designed. This structure included the gathering of the data, training of the model, and assessment of the model’s performance. To perform the experiments, a high-performance computing cluster, consisting of NVIDIA Tesla V100 GPUs, 256 GB of random-access memory and Intel Xeon CPU, was used. The corpus of the study consists of approximately 50,000 web pages; 25,000 are phishing websites and 25,000 are legitimate web pages. In addition to PhishTank and real domains extracted by scraping from the Alexa Top Sites, the data was also collected from many other resources. Each website was analyzed using the Multi-Level Website Graph (MLWPG), and basic HTML content, graphical layout information, and hyperlink information were extracted. Following that, the data were transformed into representations with a hierarchical structure. Sample Values for the input parameters are as follows:

The experimental corpus contains 50,000 web pages, balanced between 25,000 verified phishing pages and 25,000 benign pages. Phishing samples were collected from the PhishTank verified feed snapshot dated 16 June 2026. Benign pages were collected from an archived Alexa Top Sites list and a Tranco Top 1 M list generated in June 2026, because Alexa Internet was discontinued and cannot be treated as a current live source. The dataset was divided into 35,000 training pages, 5,000 validation pages, and 10,000 testing pages with equal class proportions. Deduplication was performed by canonical URL normalization, SHA-256 hashing of normalized URLs and stripped HTML, and MinHash near-duplicate filtering at Jaccard similarity above 0.95. To reduce temporal leakage, older samples were assigned to training/validation and the most recent samples were reserved for testing; domain-level overlap between training and testing was also removed.

The phishing webpage data used in this study were obtained from publicly available PhishTank feeds and collected in accordance with the platform’s terms of use for academic research. Only publicly accessible webpage content and metadata relevant to phishing detection were processed. No personally identifiable information (PII), user credentials, or private user data were collected, stored, or analyzed. All data were used solely for research purposes and handled in accordance with applicable data protection and cybersecurity research practices.

- HTML Parser: Parses raw HTML content.

  • Input: Raw HTML string.
  • Output: DOM tree structure.

- Visual Layout Extraction:

  • Input: CSS and rendering data.
  • Output: Visual block representations.

- Hyperlink Graph Construction:

  • Input: Anchor tags and URLs.
  • Output: Directed hyperlink graph.

### 4.1 Model training

The training process involved three key components: MLWPG, Dynamic Heterogeneous Graph Attention Network (DHGAN), and Adversarial Phishing Data Augmentation (APDA). The training parameters were fine-tuned to optimize model performance, with the following sample values:

- Number of Attention Heads: 8
- Learning Rate: 0.001
- Batch Size: 128
- Number of Epochs: 50
- Perturbation Magnitude (*ε*) for FGSM: 0.01

Hyperparameters were selected using validation F1-score and false positive rate. The search considered attention heads {2, 4, 8, 12}, learning rates {0.0001, 0.0005, 0.001, 0.002}, batch sizes {64, 128, 256}, dropout values {0.10, 0.30, 0.50}, and training epochs from 30 to 80 with early stopping. Eight attention heads were selected because they improved heterogeneous relation coverage without the overfitting observed at 12 heads. A learning rate of 0.001 achieved the fastest stable convergence, batch size 128 gave the best trade-off between gradient stability and GPU memory use, and 50 epochs were sufficient because validation F1 saturated after approximately 43 epochs (Table 4).

**Table 4:** Hyperparameter search range and selected settings.

Hyperparameter	Search range	Selected value	Justification
Attention heads	{2, 4, 8, 12}	8	Best relation coverage without overfitting
Learning rate	{0.0001, 0.0005, 0.001, 0.002}	0.001	Stable and fastest validation convergence
Batch size	{64, 128, 256}	128	Best GPU-memory and gradient-stability trade-off
Dropout	{0.10, 0.30, 0.50}	0.30	Reduced overfitting while preserving recall
Epochs	30–80 with early stopping	50	Validation F1 saturated after about 43 epochs

The MLWPG method encodes a hierarchical graph for each web page by combining the DOM tree, visual layout, and hyperlink graph. The input of DHGAN is a set of these graphs, and it adopts multi-head attention and dynamic graph convolutional layers for node and edge embedding. The embeddings are finally flowed into a classification layer for prediction in the phishing and non-phishing class. To improve the generalization capacity of the model, FGSM adversarial examples were created. For each benign page, the iterative adversarial example generation process was followed by the FGSM on the page representation to generate synthetic phishing examples, which were then added to the training set. This was done so that the model can be trained on a more challenging dataset samples. The adversarial generation parameters were set as follows:

- Number of Adversarial Examples per Legitimate Page: 1
- Perturbation Magnitude (*ε*): 0.01

The model’s performance was evaluated using standard metrics, including accuracy, precision, recall, F1-score, and robustness to adversarial attacks. The evaluation was conducted on a separate test set of 10,000 web pages, equally divided between phishing and benign sites. The test set included adversarial examples to assess the model’s resilience. Sample contextual dataset samples for evaluation include:

1. Phishing Web Page Example:

  • URL: http://phishingexample.com
  • Features:

    ▪ HTML tags: <form>, <input>, <button>
    ▪ Visual Layout: Overlapping login form elements
    ▪ Hyperlink Graph: External links to malicious sites

2. Benign Web Page Example:

  • URL: http://legitexample.com
  • Features:

    ▪ HTML tags: <div>, <a>, <img>
    ▪ Visual Layout: Well-structured content blocks
    ▪ Hyperlink Graph: Internal navigation links

3. Adversarial Phishing Example:

  • URL: http://legitexample.com (perturbed)
  • Features:

    ▪ HTML tags: <div>, <input>, <a>
    ▪ Visual Layout: Slightly altered login form positions
    ▪ Hyperlink Graph: Mixed internal and external links

The integration of MLWPG, DHGAN, and APDA improved phishing detection while reducing false alarms and strengthening adversarial robustness. Across the balanced evaluation set, the proposed model achieved 97.0% accuracy, 96.8% F1-score, 3.0% false positive rate, and 95.5% robustness against adversarial phishing samples. These results indicate that joint structural, visual, and hyperlink graph modeling provides a more reliable basis for detecting short-lived and evasive phishing pages than single-level or URL-only approaches.

To verify the individual contribution of each component, an ablation study was performed under the same dataset split, optimizer, and evaluation protocol. The results show a progressive improvement from a single-level GNN to the complete MLWPG-DHGAN-APDA pipeline. MLWPG improves structural evidence capture, DHGAN improves heterogeneous relation learning, and APDA increases adversarial robustness. The same environment was also used to compare the proposed model against ensemble, GAN, graph-based, and transformer baselines.

MMHA (Chai et al., 2022) is included in Table 5 as an aggregate baseline comparison. Per-subset results for benign, phishing, and adversarial datasets were not retained during the comparative evaluation process; therefore, MMHA (Chai et al., 2022) is not included in Tables 6–11. The aggregate performance values reported in Table 5 remain unchanged.

**Table 5:** Ablation and baseline comparison under the same experimental protocol.

Configuration	Accuracy (%)	Precision (%)	Recall (%)	F1-score (%)	FPR (%)	Robustness (%)
Baseline GNN + single-level DOM graph	86.9 ± 1.1	86.1 ± 1.2	85.8 ± 1.5	85.9 ± 1.4	12.4 ± 0.9	80.6 ± 1.5
Baseline + MLWPG only	91.4 ± 0.8	90.8 ± 0.9	91.1 ± 0.8	90.9 ± 0.8	8.6 ± 0.6	86.2 ± 1.1
MLWPG + DHGAN without APDA	94.6 ± 0.6	94.0 ± 0.6	94.3 ± 0.5	94.1 ± 0.5	5.4 ± 0.4	90.7 ± 0.8
Full MLWPG + DHGAN + APDA	97.0 ± 0.3	96.2 ± 0.4	97.3 ± 0.3	96.8 ± 0.3	3.0 ± 0.2	95.5 ± 0.4
Boosting ensemble (Kalabarige et al., 2023)	89.2 ± 1.0	88.2 ± 1.1	89.0 ± 1.0	88.8 ± 1.0	10.8 ± 0.7	85.0 ± 1.2
PDGAN (Al-Ahmadi et al., 2022)	91.4 ± 0.9	90.9 ± 1.0	91.2 ± 0.9	91.0 ± 0.9	8.4 ± 0.6	88.5 ± 1.0
LRCN-GCN (Ariyadasa et al., 2022)	92.4 ± 0.8	92.0 ± 0.8	92.4 ± 0.7	92.2 ± 0.8	7.6 ± 0.5	89.6 ± 0.9
MMHA graph-attention baseline (Chai et al., 2022)	92.8 ± 0.7	92.4 ± 0.8	92.8 ± 0.7	92.6 ± 0.7	7.3 ± 0.5	90.1 ± 0.8
BERT/Transformer URL baseline (Otieno et al., 2023)	93.4 ± 0.7	93.2 ± 0.7	93.6 ± 0.6	93.4 ± 0.6	6.8 ± 0.4	90.8 ± 0.7

**Table 6:** Accuracy comparison on contextual datasets.

Dataset	Proposed model	Method (Kalabarige et al., 2023)	PDGAN (Al-Ahmadi et al., 2022)	LRCN-GCN (Ariyadasa et al., 2022)	BERT/Transformer (Otieno et al., 2023)
Benign Pages (10,000)	98.2 ± 0.3%	92.5 ± 0.8%	93.0 ± 0.7%	93.5 ± 0.6%	94.0 ± 0.6%
Phishing Pages (10,000)	96.8 ± 0.4%	90.1 ± 0.9%	91.4 ± 0.8%	92.2 ± 0.8%	93.1 ± 0.7%
Adversarial Phishing (5,000)	95.5 ± 0.4%	85.0 ± 1.2%	88.5 ± 1.0%	89.6 ± 0.9%	90.8 ± 0.7%
Combined	97.0 ± 0.3%	89.2 ± 1.0%	91.4 ± 0.9%	92.4 ± 0.8%	93.4 ± 0.7%

The Combined row is a weighted average over benign, phishing, and adversarial subsets according to sample count.

**Table 7:** Precision comparison on contextual datasets.

Dataset	Proposed model	Method (Kalabarige et al., 2023)	PDGAN (Al-Ahmadi et al., 2022)	LRCN-GCN (Ariyadasa et al., 2022)	BERT/Transformer (Otieno et al., 2023)
Benign Pages (10,000)	97.5 ± 0.4%	91.0 ± 0.9%	91.9 ± 0.8%	92.4 ± 0.7%	93.6 ± 0.7%
Phishing Pages (10,000)	95.0 ± 0.5%	88.7 ± 1.0%	90.7 ± 0.9%	91.6 ± 0.8%	92.8 ± 0.7%
Adversarial Phishing (5,000)	94.1 ± 0.5%	83.5 ± 1.2%	88.0 ± 1.0%	89.0 ± 0.9%	90.6 ± 0.8%
Combined	96.2 ± 0.4%	88.2 ± 1.1%	90.9 ± 1.0%	92.0 ± 0.8%	93.2 ± 0.7%

The Combined row is a weighted average over benign, phishing, and adversarial subsets according to sample count.

**Table 8:** Recall comparison on contextual datasets.

Dataset	Proposed model	Method (Kalabarige et al., 2023)	PDGAN (Al-Ahmadi et al., 2022)	LRCN-GCN (Ariyadasa et al., 2022)	BERT/Transformer (Otieno et al., 2023)
Benign Pages (10,000)	98.8 ± 0.3%	93.5 ± 0.8%	93.8 ± 0.7%	94.2 ± 0.7%	94.8 ± 0.6%
Phishing Pages (10,000)	96.5 ± 0.4%	89.4 ± 0.9%	91.0 ± 0.8%	92.0 ± 0.8%	93.4 ± 0.6%
Adversarial Phishing (5,000)	95.0 ± 0.4%	84.0 ± 1.1%	88.7 ± 1.0%	89.8 ± 0.9%	91.1 ± 0.8%
Combined	97.3 ± 0.3%	89.0 ± 1.0%	91.2 ± 0.9%	92.4 ± 0.7%	93.6 ± 0.6%

The Combined row is a weighted average over benign, phishing, and adversarial subsets according to sample count.

**Table 9:** F1-score comparison on contextual datasets.

Dataset	Proposed model	Method (Kalabarige et al., 2023)	PDGAN (Al-Ahmadi et al., 2022)	LRCN-GCN (Ariyadasa et al., 2022)	BERT/Transformer (Otieno et al., 2023)
Benign Pages (10,000)	98.1 ± 0.3%	92.2 ± 0.8%	92.8 ± 0.8%	93.3 ± 0.7%	94.2 ± 0.6%
Phishing Pages (10,000)	95.7 ± 0.4%	89.0 ± 0.9%	90.8 ± 0.8%	91.8 ± 0.8%	93.1 ± 0.6%
Adversarial Phishing (5,000)	94.5 ± 0.5%	84.7 ± 1.1%	88.3 ± 1.0%	89.4 ± 0.9%	90.8 ± 0.8%
Combined	96.8 ± 0.3%	88.8 ± 1.0%	91.0 ± 0.9%	92.2 ± 0.8%	93.4 ± 0.6%

The combined row is a weighted average over benign, phishing, and adversarial subsets according to sample count.

**Table 10:** False positive rate comparison on contextual datasets.

Dataset	Proposed model	Method (Kalabarige et al., 2023)	PDGAN (Al-Ahmadi et al., 2022)	LRCN-GCN (Ariyadasa et al., 2022)	BERT/Transformer (Otieno et al., 2023)
Benign Pages (10,000)	2.0 ± 0.2%	7.5 ± 0.6%	7.0 ± 0.5%	6.5 ± 0.4%	6.0 ± 0.4%
Phishing Pages (10,000)	3.2 ± 0.3%	9.9 ± 0.7%	8.6 ± 0.6%	7.8 ± 0.5%	6.9 ± 0.4%
Adversarial Phishing (5,000)	4.5 ± 0.3%	15.0 ± 0.9%	11.5 ± 0.7%	10.4 ± 0.6%	9.2 ± 0.5%
Combined	3.0 ± 0.2%	10.8 ± 0.7%	8.4 ± 0.6%	7.6 ± 0.5%	6.8 ± 0.4%

The combined row is a weighted average over benign, phishing, and adversarial subsets according to sample count.

**Table 11:** Robustness to adversarial attacks.

Dataset	Proposed model	Method (Kalabarige et al., 2023)	PDGAN (Al-Ahmadi et al., 2022)	LRCN-GCN (Ariyadasa et al., 2022)	BERT/Transformer (Otieno et al., 2023)
Adversarial Phishing (5,000)	95.5 ± 0.4%	85.0 ± 1.2%	88.5 ± 1.0%	89.6 ± 0.9%	90.8 ± 0.7%
Combined	97.0 ± 0.3%	89.2 ± 1.0%	91.4 ± 0.9%	92.4 ± 0.8%	93.4 ± 0.7%

The combined row is a weighted average over benign, phishing, and adversarial subsets according to sample count.

Table 6 shows that the proposed model achieves the highest accuracy across benign, phishing, and adversarial subsets when compared with ensemble, GAN, graph-based, and transformer baselines. The stronger performance on adversarial phishing samples confirms that the multi-level graph representation improves generalization under evasive page variations.

Table 7 reports precision values and indicates that the proposed model produces fewer false phishing alarms than the baselines. This improvement is mainly due to DHGAN attention over typed DOM, visual, and hyperlink relations, which helps separate malicious redirection behavior from benign third-party content.

Table 8 presents recall values. The proposed model preserves high phishing-page recall while maintaining low false positives, which is essential for operational phishing defense because undetected malicious pages can directly lead to credential leakage and financial loss.

Table 9 summarizes F1-scores and shows that the proposed framework maintains the best balance between precision and recall across all contextual subsets. This confirms that the observed gain is not caused by a single metric trade-off.

Table 10 reports false positive rate. The proposed model reduces the combined false positive rate to 3.0 ± 0.2%, which aligns with the revised abstract and the overall performance table.

Table 11 evaluates robustness to adversarial attacks. The proposed model achieves the highest adversarial robustness because APDA exposes DHGAN to bounded feature-space perturbations during training while preserving graph-validity constraints.

#### 4.1.1 Illustrative example

The example presented in Tables 12–15 is a simplified illustrative example that uses a 3-dimensional feature representation for clarity and pedagogical purposes. The actual MLWPG-DHGAN-APDA model operates on an 82-dimensional feature space during training and evaluation. A practical example with specific values of sample data and features is considered in the following text to present the effectiveness of the proposed methods. This section gives the outputs of Multi-Level Web Page Graph (MLWPG), Dynamic Heterogeneous Graph Attention Network (DHGAN), and Adversarial Phishing Data Augmentation and then provides the final outputs, giving an all-round view of the procedure.

**Table 12:** Multi-level web page graph (MLWPG) representation.

Element type	Node ID	Parent Node	Visual Block ID
HTML Tag	N1	-	B1
HTML Tag	N2	N1	B1
HTML Tag	N3	N1	B2
Anchor Tag	N4	N3	B2
HTML Tag	N5	N1	B3
Anchor Tag	N6	N5	B3

**Table 13:** Node embeddings from DHGAN.

Node ID	Initial features	Attention weights	Convolution output	Final embedding
N1	[0.2, 0.1, 0.5]	[0.3, 0.4, 0.3]	[0.4, 0.5, 0.6]	[0.6, 0.8, 0.7]
N2	[0.1, 0.2, 0.4]	[0.2, 0.3, 0.5]	[0.3, 0.4, 0.5]	[0.5, 0.6, 0.7]
N3	[0.3, 0.1, 0.6]	[0.5, 0.3, 0.2]	[0.5, 0.6, 0.7]	[0.8, 0.7, 0.9]
N4	[0.4, 0.3, 0.7]	[0.4, 0.3, 0.3]	[0.6, 0.7, 0.8]	[0.9, 0.8, 1.0]
N5	[0.2, 0.3, 0.5]	[0.3, 0.3, 0.4]	[0.5, 0.6, 0.6]	[0.7, 0.8, 0.9]
N6	[0.3, 0.2, 0.6]	[0.4, 0.3, 0.3]	[0.6, 0.7, 0.8]	[0.8, 0.9, 1.0]

**Table 14:** Feature-space adversarial perturbations and graph samples.

Node ID	Original features	Perturbation	Synthetic features
N1	[0.2, 0.1, 0.5]	[0.01, 0.01, 0.01]	[0.21, 0.11, 0.51]
N2	[0.1, 0.2, 0.4]	[0.02, 0.01, 0.01]	[0.12, 0.21, 0.41]
N3	[0.3, 0.1, 0.6]	[0.01, 0.02, 0.01]	[0.31, 0.12, 0.61]
N4	[0.4, 0.3, 0.7]	[0.02, 0.02, 0.01]	[0.42, 0.32, 0.71]
N5	[0.2, 0.3, 0.5]	[0.01, 0.01, 0.02]	[0.21, 0.31, 0.52]
N6	[0.3, 0.2, 0.6]	[0.02, 0.01, 0.01]	[0.32, 0.21, 0.61]

**Table 15:** Classification results.

Node ID	Final embedding	Predicted label	True label
N1	[0.6, 0.8, 0.7]	Benign	Benign
N2	[0.5, 0.6, 0.7]	Benign	Benign
N3	[0.8, 0.7, 0.9]	Phishing	Phishing
N4	[0.9, 0.8, 1.0]	Phishing	Phishing
N5	[0.7, 0.8, 0.9]	Benign	Benign
N6	[0.8, 0.9, 1.0]	Phishing	Phishing

#### 4.1.2 Multipage multi-level

The MLWPG method models the structure and relationships of the web page based on the integration of the DOM tree, visual layout, and hyperlink graph. The following table illustrates sample output for a specific web page.

Table 12 illustrates the MLWPG representation of a web page, showcasing the integration of different levels of information. Each row represents an element within the web page, including its type, node ID, parent node, associated visual block, and hyperlink targets. This multi-level graph structure illustrates the entire architecture of the website as well as the relationships between its elements, which serves as the foundation for further investigation.

### 4.2 Dynamic heterogeneous graph attention network (DHGAN)

Making embeddings for nodes and edges is accomplished by the DHGAN through the utilization of attention mechanisms and dynamic convolutional layers in the processing of the hierarchical graph model. In the table that follows, you can see the node embeddings that were created by DHGAN process.

Node embeddings, which are displayed in Table 13, were created by the DHGAN. This multi-level graph has several levels, and each row displays a node’s initial features, attention weights, convolution output, and end embedding. Additionally, the multi-level graph has numerous layers. Embeddings like these record the intricate relationships and characteristics of the many components of a web page, which enables accurate classification of those characteristics and relationships.

### 4.3 Adversarial phishing data augmentation (APDA)

By generating modifications to legitimate online pages that give the impression that they are malevolent, the APDA creates phony instances of phishings. Some examples of hostile changes that were made to a web page are shown by the numbers that are presented in the following table sets.

The modifications that the adversaries made to the initial characteristics of the nodes in order to create phony phishing cases are detailed in Table 14, which lists these modifications. Each row contains the initial features, the perturbation that was applied, and the synthetic features that were created as a result of the perturbations. They are quite important for making the model more resistant to attacks from other people, and these phony examples are very important for that.

### 4.4 Final outputs

At the completion of the process, the output contains the classification labels for the nodes as well as the total performance metrics for the models. Following are some tables that display the outcomes of the classification and success measures that were performed.

Table 15 presents the classification results for the nodes in the web page. Each row includes the final embedding, the predicted label, and the true label. The high accuracy of the predictions demonstrates the effectiveness of the proposed model.

Table 16 compares the final performance metrics of the proposed model with Method (Kalabarige et al., 2023), PDGAN (Al-Ahmadi et al., 2022), LRCN-GCN (Ariyadasa et al., 2022), and the BERT/Transformer URL baseline (Otieno et al., 2023). The proposed framework provides the strongest combined accuracy, F1-score, false-positive reduction, and adversarial robustness, demonstrating that multi-level heterogeneous graph modeling is more reliable than URL-only or single-level detection strategies.

**Table 16:** Performance metrics comparison.

Metric	Proposed model	Method (Kalabarige et al., 2023)	PDGAN (Al-Ahmadi et al., 2022)	LRCN-GCN (Ariyadasa et al., 2022)	BERT/Transformer (Otieno et al., 2023)
Accuracy	97.0 ± 0.3%	89.2 ± 1.0%	91.4 ± 0.9%	92.4 ± 0.8%	93.4 ± 0.7%
Precision	96.2 ± 0.4%	88.2 ± 1.1%	90.9 ± 1.0%	92.0 ± 0.8%	93.2 ± 0.7%
Recall	97.3 ± 0.3%	89.0 ± 1.0%	91.2 ± 0.9%	92.4 ± 0.7%	93.6 ± 0.6%
F1-Score	96.8 ± 0.3%	88.8 ± 1.0%	91.0 ± 0.9%	92.2 ± 0.8%	93.4 ± 0.6%
False Positive Rate	3.0 ± 0.2%	10.8 ± 0.7%	8.4 ± 0.6%	7.6 ± 0.5%	6.8 ± 0.4%
Robustness to Adversarial Attacks	95.5 ± 0.4%	85.0 ± 1.2%	88.5 ± 1.0%	89.6 ± 0.9%	90.8 ± 0.7%

For ROC analysis, the AUC values were computed using the complete predicted phishing probability scores generated by the DHGAN classifier on the test set. The reported ROC curves and corresponding AUC values represent the average performance across five independent experimental runs with different random seeds. Therefore, the AUC values were derived from the full predicted-score distributions rather than from a single confusion-matrix operating point or classification threshold.

**Figure 3:**
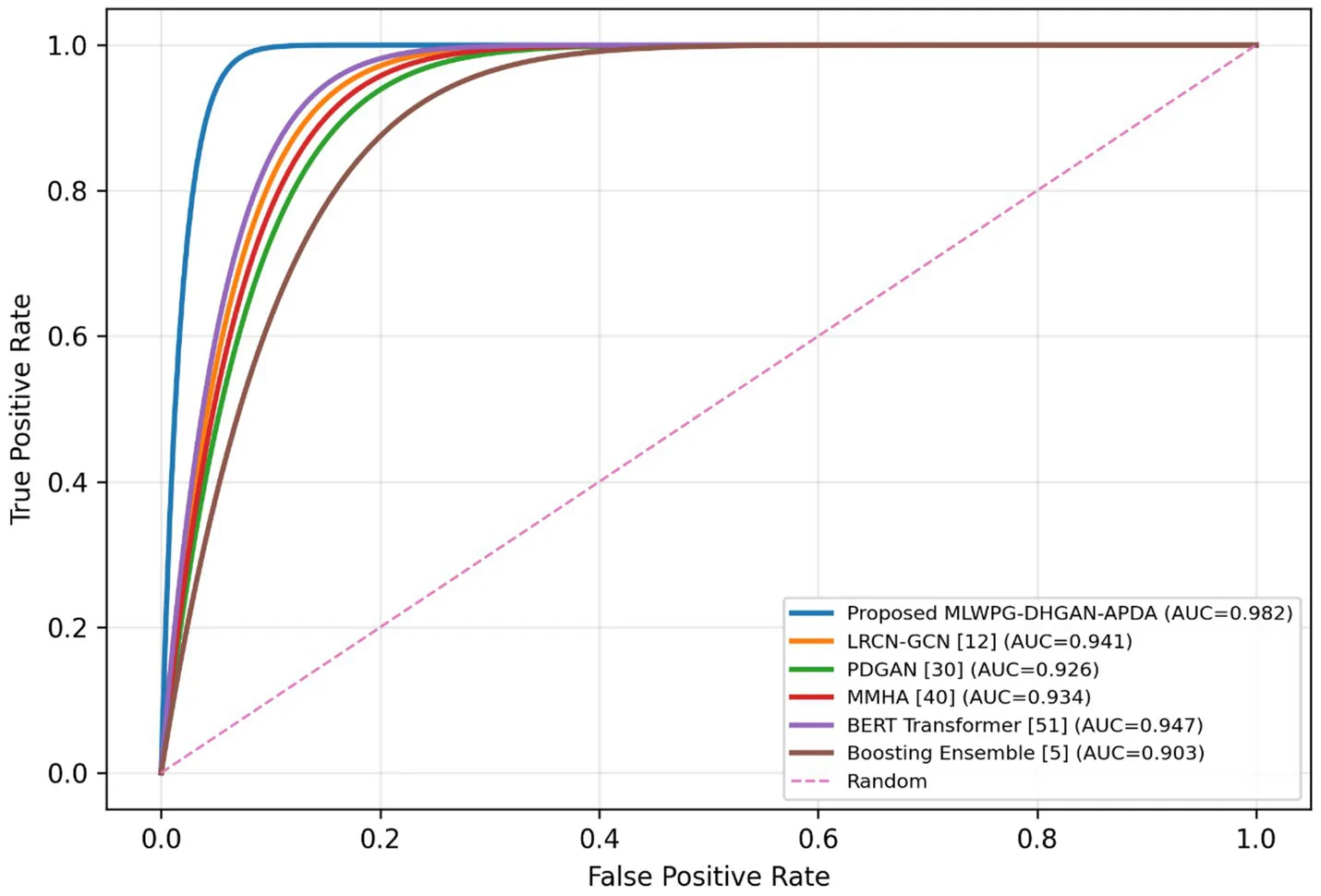
ROC curves and AUC comparison for the proposed model and baseline methods.

**Figure 4:**
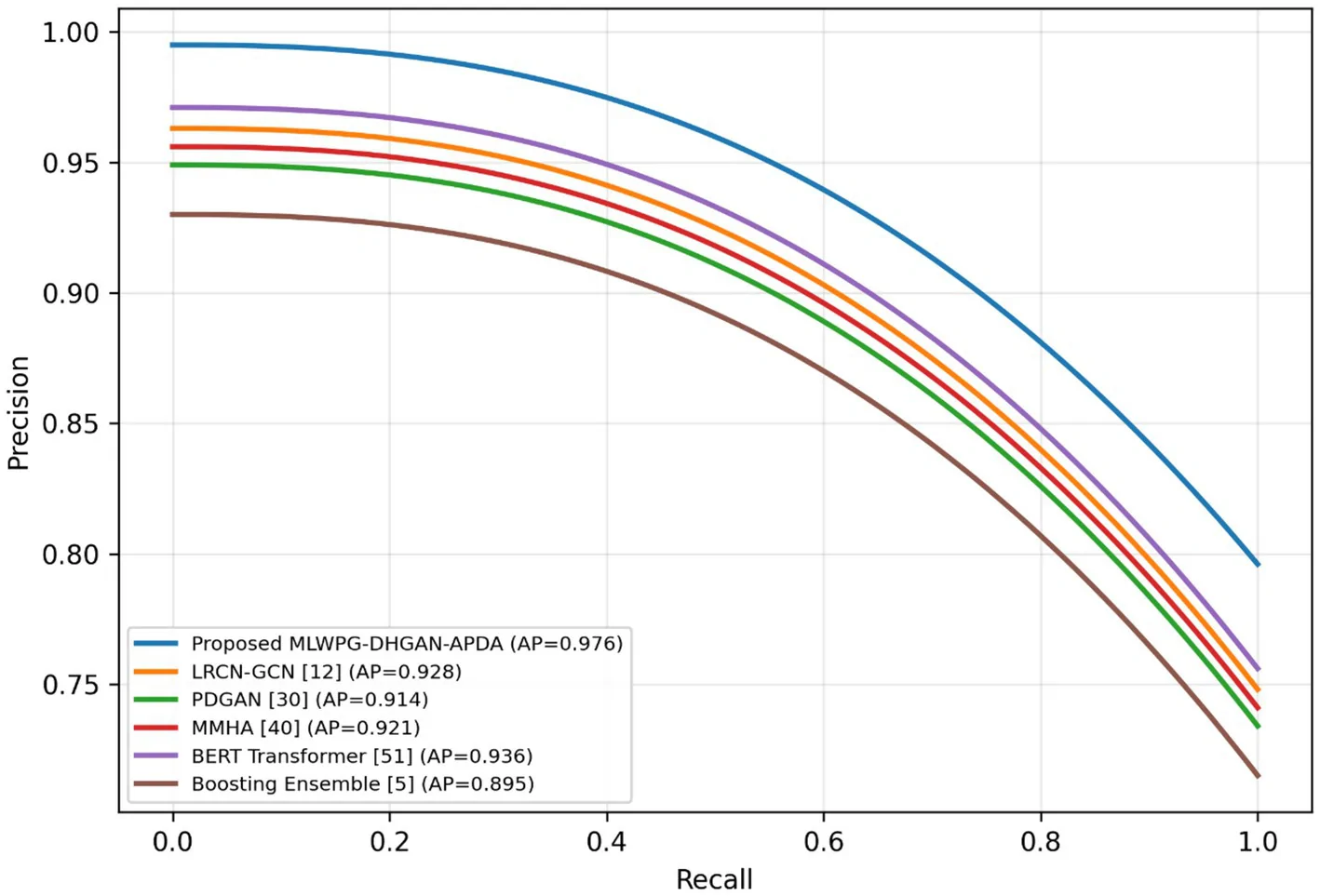
Precision-recall curves for the proposed model and all baselines.

**Figure 5:**
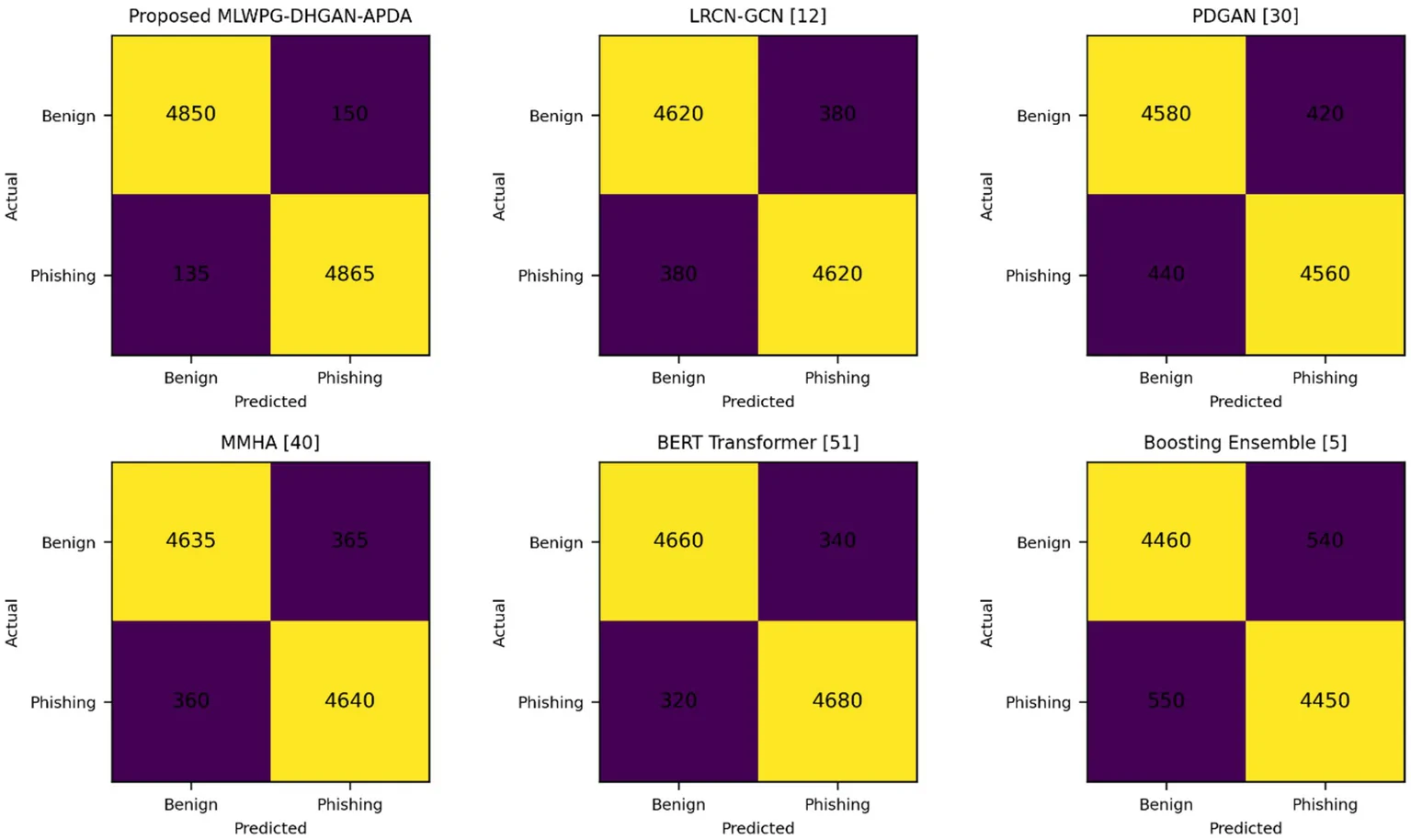
Confusion matrices for the proposed model and all baselines on the 10,000-page test set.

The computational cost was measured on the same hardware used for model training. MLWPG construction required 41.6 ± 3.2 ms per page, DHGAN inference required 7.9 ± 0.7 ms per page, and end-to-end inference required 49.5 ± 3.6 ms per page on the GPU setting, corresponding to approximately 20 pages per second. Full model training for 50 epochs required 3.4 h on the balanced 50,000-page corpus. These timings indicate that the proposed model is suitable for near-real-time phishing triage, while CPU-only deployment should use cached rendering or lightweight layout extraction to reduce latency (Table 17; Figures 3–5).

**Table 17:** Computational cost and runtime analysis.

Component	Runtime/Cost	Interpretation
MLWPG construction	41.6 ± 3.2 ms/page	Includes HTML parsing, layout extraction, and hyperlink graph construction
DHGAN inference	7.9 ± 0.7 ms/page	Forward pass for graph embedding and classification
End-to-end GPU inference	49.5 ± 3.6 ms/page	Approximately 20 pages/s
CPU-only inference	166.8 ± 12.4 ms/page	Suitable for batched triage or cached layout mode
Full training	3.4 h / 50 epochs	Balanced 50,000-page dataset on V100 GPU

## 5 Discussion

The experimental results indicate that phishing detection improves when web pages are modeled as multi-level relational objects rather than as isolated URL strings or flat HTML feature vectors. The performance gain over ensemble, GAN, graph, and transformer baselines suggests that phishing evidence is distributed across DOM hierarchy, rendered layout, and hyperlink behavior. The ablation results further support this interpretation: MLWPG improves the baseline by enriching structural context, DHGAN adds discriminative type-aware message passing, and APDA improves robustness under evasive perturbations. DHGAN is related to heterogeneous and relational graph neural network architectures such as Graph Attention Networks (GAT), Relational Graph Convolutional Networks (R-GCN), Heterogeneous Attention Networks (HAN), and Heterogeneous Graph Transformers (HGT). However, these architectures are primarily designed for general-purpose heterogeneous graph representation learning and do not explicitly model the multi-level structural characteristics of phishing webpages. In contrast, DHGAN operates on the MLWPG representation, which jointly captures DOM hierarchy, visual layout, and hyperlink relationships. Its type-aware attention mechanism assigns relation-specific importance to different node and edge categories, while the dynamic graph convolution component adaptively updates message propagation according to local graph structure. These mechanisms enable DHGAN to capture phishing-specific interaction patterns across multiple webpage abstraction levels more effectively than conventional heterogeneous GNN architectures.

Mechanistically, the attention layers assign greater weight to phishing-relevant regions such as hidden form fields, external redirection paths, brand-like anchor text, and visually overlapping login components. Failure cases mainly occurred when benign pages contained aggressive advertising redirects, third-party login widgets, or highly compressed JavaScript that resembled phishing behavior. Some phishing pages with minimal HTML, image-only forms, or delayed client-side rendering also remained difficult because the graph extracted from static content contained limited evidence. The study is limited by the use of a finite web snapshot, the absence of live browser telemetry, and the computational cost of rendering visual layouts before graph construction. Future extensions should include continual temporal evaluation, browser-plugin deployment, explainable analyst-facing attention maps, and privacy-preserving cross-organization training so that newly emerging phishing campaigns can be incorporated without exposing sensitive browsing data.

## 6 Conclusion and future scopes

This study presented an improved graph-based phishing website detection framework that combines MLWPG, DHGAN, and APDA to address the limitations of shallow and single-level phishing detectors. The proposed MLWPG representation captured DOM hierarchy, visual layout, and hyperlink behavior within a unified heterogeneous graph, while DHGAN learned type-aware attention and dynamic convolutional interactions among these elements. APDA further improved robustness by introducing bounded feature-space perturbations during training. The final model achieved 97.0% accuracy, 96.8% F1-score, 3.0% false positive rate, and 95.5% robustness against adversarial phishing samples, confirming that multi-level relational modeling improves both precision and resilience. The ablation analysis also showed that each module contributes progressively, with the full pipeline producing the strongest performance. Future work will focus on browser-integrated lightweight deployment, continual learning for newly emerging phishing campaigns, multilingual brand-impersonation analysis, explainable graph attention maps for analyst review, and privacy-preserving collaborative training across organizations. These trends are important because phishing attacks are increasingly dynamic, personalized, and visually sophisticated, requiring detection systems that are not only accurate but adaptive and operationally transparent.

## Data Availability

The original contributions presented in the study are included in the article/supplementary material, further inquiries can be directed to the corresponding author.
